# Biofilm formation by *Bacillus subtilis*: new insights into regulatory strategies and assembly mechanisms

**DOI:** 10.1111/mmi.12697

**Published:** 2014-07-18

**Authors:** Lynne S Cairns, Laura Hobley, Nicola R Stanley-Wall

**Affiliations:** 1 Division of Molecular Microbiology, College of Life Sciences, University of DundeeDundee, DD1 5EH, UK

## Abstract

Biofilm formation is a social behaviour that generates favourable conditions for sustained survival in the natural environment. For the Gram-positive bacterium *Bacillus subtilis* the process involves the differentiation of cell fate within an isogenic population and the production of communal goods that form the biofilm matrix. Here we review recent progress in understanding the regulatory pathways that control biofilm formation and highlight developments in understanding the composition, function and structure of the biofilm matrix.

## Introduction

It is now recognized that the majority of microbes live in complex sessile communities called biofilms. In the biofilm individual cells are held together by a self-produced extracellular polymeric matrix commonly comprised of polysaccharides, proteins and DNA (Branda *et al*., 2005). For microbes, the biofilm lifestyle confers several advantages. For example, inhabitants can access hard to reach nutrients and receive protection from fluctuations in environmental conditions (Costerton *et al*., 1995). Indeed, due to these properties, biofilms have been harnessed in industrial settings for bioremediation purposes (Halan *et al*., 2012) and additionally exhibit the potential to be used in agricultural settings as a biological alternative to petrochemical derived fertilizers (Bais *et al*., 2004). However, one corollary is that biofilms frequently present problems with regard to public health, particularly due to their ability to colonize both natural and artificial surfaces within the human body which can result in chronic infections (Hall *et al*., 2014). Likewise, biofilms are problematic in industrial settings where their formation in cooling towers and pipelines, for example, can have serious implications (Liu *et al*., 2009).

While most natural biofilms are polymicrobial in composition, a great deal has been learnt, and remains to be discovered, from the analysis of single-species biofilms. Studies to investigate the molecular basis of biofilm formation by the Gram-positive soil dwelling bacterium *Bacillus subtilis* have been extensive since the recognition of its capability to form biofilms (Branda *et al*., 2001; Hamon and Lazazzera, 2001). Undeniably, this research has revealed many fundamental principles that underpin biofilm assembly, including: how complex signalling networks are integrated during a complex multicellular process (Vlamakis *et al*., 2013), how bacteria are able to sense and respond to specific stimuli (Lopez and Kolter, 2010) and how isogenic bacterial cells differentiate to follow distinct cell lineages (Lopez *et al*., 2009). Furthermore, many details of the properties of the macromolecules that provide structure to the biofilm are now known (Romero *et al*., 2010; 2011[Bibr b67],[Bibr b68]; Kobayashi and Iwano, 2012; Hobley *et al*., 2013). Many of the concepts which stem from studies in the *B. subtilis* biofilm field have broad implications for a range of bacterial species.

**Figure 1:**
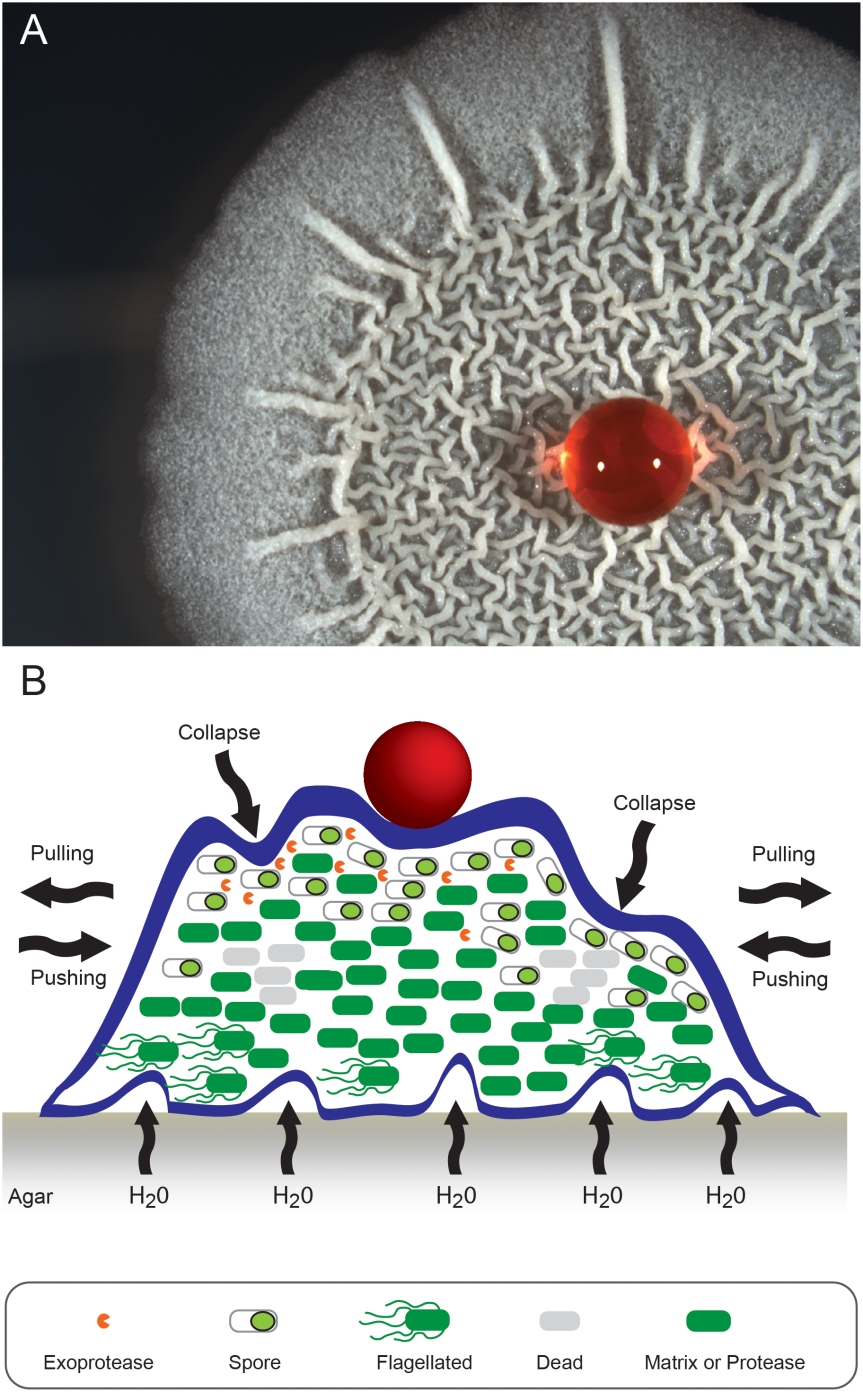
*Bacillus subtilis* biofilm formation.A. The mature biofilm exhibits a complex network of intertwined wrinkles and ridges and is highly hydrophobic. A 7 μl water droplet stained with red food colouring was placed on the biofilm.B. The mature biofilm is generated as a consequence of many converging factors. This is represented schematically in cross-section in this figure. Contributing to biofilm formation is the differentiation of cell fate in the population, the death of cells at the base of wrinkles, the mechanical forces imparted by the biofilm matrix that both push or pull the community and the production of the extracellular matrix. The BslA coat is shown as a dark blue layer and the EPS and TasA fibres encased within this boundary but are not depicted. The red ball represents a water droplet and shows the hydrophobicity exhibited by the structure. Channels that allow fluids to flow into the biofilm are shown at the base of the structure.

*B. subtilis* biofilms are predominantly studied using an ancestral strain called NCIB3610 and three experimental systems, namely: pellicle formation, where the architecturally complex bacterial community forms at an air-liquid interface (Branda *et al*., 2001), rugose colony formation on semi-solid agar surfaces (Branda *et al*., 2001) (Fig. 1A) and finally, given the role of *B. subtilis* as a biocontrol agent in agricultural settings, an increasing number of studies have focussed on the formation of biofilms on plant roots (Bais *et al*., 2004; Chen *et al*., 2012; 2013; Beauregard *et al*., 2013). A fundamental principle linking these models is that *B. subtilis* functions as a cooperative community both by differentiation of the isogenic progenitor population into specialized cell types (Vlamakis *et al*., 2008) and by the production of shared macromolecules that form the communal biofilm matrix (Branda *et al*., 2006; Ostrowski *et al*., 2011). The *B. subtilis* biofilm matrix consists of proteins called TasA and TapA (Branda *et al*., 2006; Romero *et al*., 2011) and a large molecular weight secreted polysaccharide (Branda *et al*., 2001). Assembly of the mature biofilm also requires the presence of the biofilm coat protein called BslA (formerly YuaB) (Ostrowski *et al*., 2011; Kobayashi and Iwano, 2012; Hobley *et al*., 2013). Each of these extracellular molecules has the capacity to function as a ‘communal good’ in the community and production is subject to tight transcriptional control. Many aspects of the environmental signals and the regulatory pathways that influence *B. subtilis* biofilm formation have been extensively reviewed elsewhere (Lopez *et al*., 2009; Lopez and Kolter, 2010; Vlamakis *et al*., 2013). Therefore, here we will summarize the most recent insights into the regulatory networks that control biofilm formation, and will discuss the biosynthesis and function of the macromolecules that allow the mature three-dimensional biofilm to be constructed.

## Regulating entry to biofilm formation

**Figure 2:**
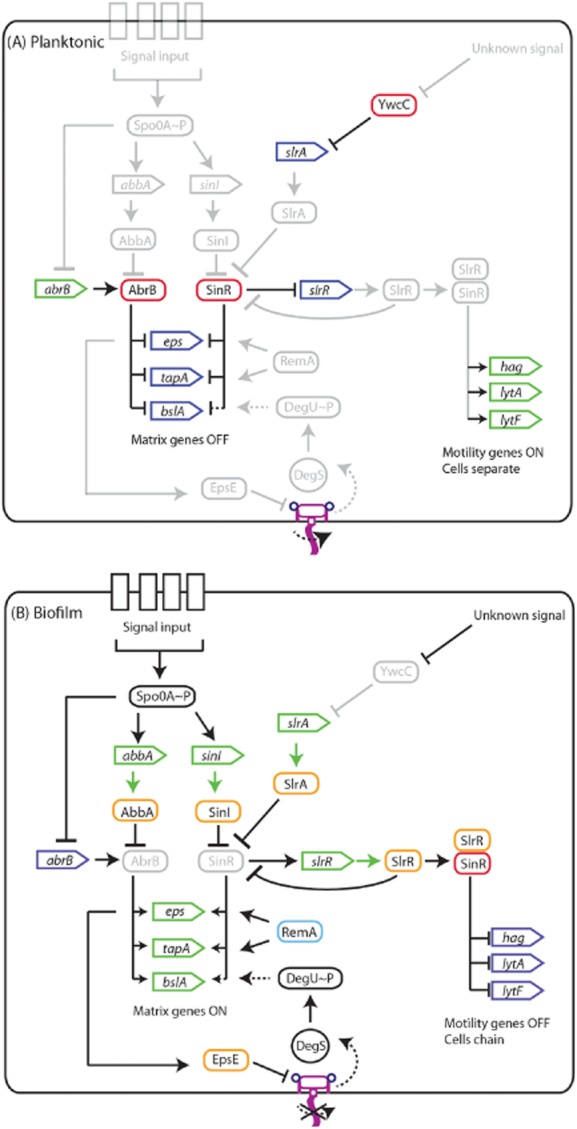
Regulatory networks governing biofilm formation. Schematic of the complex regulatory pathways that control gene transcription during (A) planktonic growth and (B) growth as a biofilm. Rounded rectangles indicate proteins, triangles indicate open reading frames (ORFs), arrows indicate activation, T-bars indicate repression. Dashed arrows or T-bars indicate indirect activation and repression respectively. Green represents active gene transcription with a green arrow indicating translation, dark blue represents absence of gene transcription, red indicates a transcriptional repressor and orange indicates a protein–protein interaction. Light blue indicates a protein that is able to bind to DNA to activate transcription. Pink structure represents a flagellum, with the curved arrow indicating rotation and the cross indicating inhibition of flagellar rotation. Vertical rectangles labelled with “signal input” indicate sensor kinases for the Spo0A pathway, for more details see Vlamakis *et al*. (2013). Faded shading indicates parts of the pathway that are inactive.

Biofilm formation is an energetically expensive process that requires the production of large macromolecules. Therefore, the decision to enter the biofilm state is tightly regulated and involves strict transcriptional control of the genes required to direct synthesis of matrix components. Phosphorylation, and thus activation, of the transcription factor Spo0A is central to biofilm initiation (Branda *et al*., 2001; Hamon and Lazazzera, 2001). Spo0A can be activated by various environmental signals that allow the cell to tune its behaviour to the local environment (Vlamakis *et al*., 2013) (Fig. 2). At threshold levels of Spo0A phosphate (Spo0A∼P) two parallel pathways of anti-repression are triggered to allow transcription of operons critical for biofilm matrix production. The first anti-repression pathway ends with removal of the transition state regulator AbrB from DNA. AbrB directly binds to DNA to repress transcription from promoters involved in a plethora of cellular processes including those needed for biofilm formation (Hamon *et al*., 2004; Banse *et al*., 2008; Chu *et al*., 2008; Kobayashi, 2008; Verhamme *et al*., 2009; Chumsakul *et al*., 2011). AbrB itself is controlled by the Spo0A pathway by two distinct means: (i) Spo0A∼P directly represses transcription of *abrB* (Strauch *et al*., 1990) and (ii) Spo0A∼P promotes the expression of *abbA*, which encodes an AbrB anti-repressor (Banse *et al*., 2008). Structural studies have recently demonstrated that AbbA functions as a DNA mimetic for AbrB binding, and thus AbbA binds to AbrB to sequester the repressor away from target DNA (Tucker *et al*., 2014) (Fig. 2).

The second anti-repression pathway revolves around the transcriptional repressor SinR which directly inhibits transcription from the 15-gene *eps* operon (required for biosynthesis of the extracellular polysaccharide) and the *tapA*-*sipW-tasA* (hereafter *tapA*) operon (Kearns *et al*., 2005; Chu *et al*., 2008). The regulatory circuitry that underpins this pathway culminates in bimodal transcription of the *eps* and *tapA* operons (Chai *et al*., 2008). The repressive effect of SinR is alleviated by an anti-repressor protein named SinI. As with production of AbbA (Banse *et al*., 2008), transcription of the *sinI* coding region is triggered by threshold levels of Spo0A∼P (Fujita *et al*., 2005). SinI binds to SinR in an essentially irreversible manner, forming a heterodimeric complex (Bai *et al*., 1993; Lewis *et al*., 1998; Scott *et al*., 1999; Newman *et al*., 2013). This leaves SinR unable to occlude target promoters, allowing transcription of target genes (Bai *et al*., 1993). Interestingly, while *sinR* is expressed in most cells, *sinI* is only transcribed by a small subpopulation (Chai *et al*., 2008). Given that a threshold level of SinI is needed to allow SinR inhibition, this leads to bimodal transcription of the *eps* and *tapA* operons (Chai *et al*., 2008). A second anti-repressor that binds to SinR, called SlrA, has also been discovered (Kobayashi, 2008; Chai *et al*., 2009). Crucially, the transcriptional regulation of *slrA* is distinct to that of *sinI*; *slrA* transcription is under the control of the transcriptional repressor YwcC, although the signal that relieves this repression is unknown (Kobayashi, 2008; Chai *et al*., 2009) (Fig. 2). However, this likely allows the integration of multiple upstream signals to repress SinR activity and activate biofilm matrix gene expression. Regulation of SinR is not restricted to transcriptional and post-translational mechanisms. Recent data link low serine levels in the cell with a decrease in the production of SinR (Subramaniam *et al*., 2013), which correspondingly triggers biofilm formation.

The action of SinR during biofilm formation is further complicated by SlrR. The *slrR* gene is under the transcriptional control of SinR and is thus expressed in the presence of high levels of SinI (Chai *et al*., 2010b). Induction of SlrR production stimulates transcription of the *tapA* and *eps* promoters (Chu *et al*., 2008; Kobayashi, 2008; Murray *et al*., 2009b), supporting the designation of SlrR as an activator of matrix production and biofilm formation (Kobayashi, 2008; Chai *et al*., 2010b). SlrR acts by binding to SinR with high affinity at equimolar stoichiometry (Chai *et al*., 2010c; Newman *et al*., 2013). This results in SinR being unable to bind to the *eps* and *tapA* promoter regions and also in the re-purposing of SinR function. The SlrR:SinR complex has unique DNA binding properties and represses transcription of genes required for motility and autolysins (Chai *et al*., 2010c). The net outcome is a concomitant repression of motility and autolysin genes with the activation of matrix genes, thereby promoting the transition from a motile state to biofilm formation (Chai *et al*., 2010c) (Fig. 2B). As SlrR re-purposes SinR activity, and prevents binding to target promoters (including that of *slrR*), it facilitates continued transcription of *slrR*. This means that cells accumulate high levels of SlrR in a self-reinforcing negative feedback loop (Chai *et al*., 2010c). In short, a cell can exist in an SlrR-low state where motility genes are expressed but matrix genes repressed, or in an SlrR-high state where cells exist as chains and are able to transcribe genes required for the synthesis of the biofilm matrix (Chai *et al*., 2010c). Fluorescent reporter fusion constructs coupled with microfluidic devices have allowed analysis of the complex network at the single cell level (Norman *et al*., 2013). Data revealed that the number of generations for which the ‘memory’ of the SlrR-high state was inherited was directly correlated with the initial level of SlrR in the cell. Basically, the higher the starting level of SlrR the greater the number of generations for which matrix gene expression was propagated (Norman *et al*., 2013).

However, removal of SinR from the *eps*, *tapA* and *slrR* promoter regions is not sufficient to allow transcription of these operons to proceed. RemA and RemB are also essential for biofilm formation (Winkelman *et al*., 2009; 2013[Bibr b86],[Bibr b87]). RemA has recently been identified as a DNA binding protein that directly activates gene expression from the *eps* and *tapA* operons, and also promotes transcription of the *slrR* gene. RemA binds directly upstream from the *eps* promoter to sites that overlap with the SinR binding sites. Therefore, SinR acts as an anti-activator to occlude RemA binding to the promoter (Winkelman *et al*., 2013). RemA also binds upstream of the *tapA* promoter, but in this instance both SinR and RemA are able to bind simultaneously (Winkelman *et al*., 2013) (Fig. 2). These studies illustrate an additional pathway by which matrix gene expression can be controlled. How RemA itself is regulated is currently unknown but, intriguingly, on the chromosome *remA* is situated alongside genes connected to the stringent response, suggesting a link between *remA* and the nutrient status of the cell (Winkelman *et al*., 2009; 2013).

## Inhibition of flagellar rotation triggers a signalling cascade

The transcription factor DegU is also intricately involved in regulating biofilm formation and exerts two opposing influences (Verhamme *et al*., 2007; Marlow *et al*., 2014b). DegU is phosphorylated by its cognate histidine kinase, DegS (Mukai *et al*., 1990). It is a pleiotropic regulator with roles in controlling many multicellular processes, including: swimming and swarming motility, biofilm formation, exoprotease production, γ-poly-d-l-glutamic acid production and sporulation (Murray *et al*., 2009a). The level of phosphorylated DegU (DegU∼P) in the cell dictates which behaviour manifests. For example, activation of biofilm formation requires intermediate-levels of DegU∼P and inhibition of biofilm formation requires high levels of DegU∼P. Biofilm activation occurs when DegU∼P indirectly promotes transcription of *bslA*, which encodes a hydrophobic biofilm coat protein (Kobayashi, 2007; Ostrowski *et al*., 2011; Hobley *et al*., 2013) (Figs 1B and 2). However, under conditions where DegU∼P levels in the cell are high, biofilm formation is inhibited due to a lack of transcription from the *eps* and *tapA* operons (Verhamme *et al*., 2007; Marlow *et al*., 2014b). In this scenario a high percentage of the cells in the community enter the sporulation pathway (Marlow *et al*., 2014b). As sporulation is a developmental process that is triggered by high levels of Spo0A∼P, these data suggest an as yet undefined link between high DegU∼P levels and high Spo0A∼P levels (Marlow *et al*., 2014b).

Several regulatory pathways have been identified as capable of controlling the level of DegU∼P in the cell (Murray *et al*., 2009a); however, a definitive signalling molecule remains enigmatic. Recent work has indicated that the DegS-DegU pathway is activated by inhibition of flagellar rotation, as may conceivably occur when a cell senses a surface prior to adherence (Cairns *et al*., 2013; Chan *et al*., 2014). Indeed, perturbation of flagellar rotation by genetic or physical means triggered an increase in DegU∼P levels. These findings suggest that upon sensing a surface DegS phosphorylates DegU to promote transcription of target genes, including *bslA*. In this way the arrest of flagellar rotation acts as an additional signal to initiate matrix synthesis (Cairns *et al*., 2013) (Fig. 2). Indeed, as will be described later, exopolysaccharide synthesis is intimately linked with a cessation of flagellar rotation, thus the cell has a mechanism to co-ordinate production of distinct components needed for biofilm assembly.

## Cell differentiation during biofilm formation

Once biofilm formation has been initiated, the assembly and maturation process can begin. Biofilm formation begins with an isogenic population of progenitor cells. As the biofilm matures the resident cells differentiate to generate multiple cell types (Fig. 1B). Differentiation was first noted after macroscopic examination of the biofilm where a sporulation specific transcriptional reporter fusion was found to be expressed in aerial tips of the developing biofilm (Branda *et al*., 2001). Subsequent data derived from fluorescent cytological reporter fusions supported this conclusion (Veening *et al*., 2006). These initial findings were built upon and, using a combination of genetics and microscopy, individual cells were shown to follow a defined developmental programme that saw a motile cell become a matrix producer that terminally differentiated into a spore forming cell (Vlamakis *et al*., 2008). Consistent with this, all three cell types can be visualized within the biofilm at distinct locations and at distinct times, suggesting spatiotemporal regulation (Vlamakis *et al*., 2008). Further work has demonstrated that cells expressing genes required for extracellular protease production can also be found in the biofilm, and that they accumulate as the biofilm matures (Marlow *et al*., 2014a). Single cell time-lapse microscopy revealed that protease producing cells arise from cells that transcribe matrix genes and showed that both cell states can coexist over multiple generations (Marlow *et al*., 2014a). These findings infer that protease production may present an additional step in cell differentiation during biofilm maturation (Marlow *et al*., 2014a). It will be of interest to establish if protease producing cells transition into environmentally resistant spores or if they remain in the protease producing state and service the community in an altruistic manner through nutrient production and possibly by degradation of the extracellular biofilm matrix. It should be noted that highly comparable cellular differentiation events have been identified in the related entomopathogen *Bacillus thuringiensis* where defined cell fates have been observed during biofilm formation (Fagerlund *et al*., 2014). However, it remains to be unequivocally established if the cell differentiation events observed constitute developmental processes *per se* or if they are a consequence of a regulatory response to environmental change and fluctuations in, for example, oxygen and nutrient levels, within the three dimensional structure of the biofilm (Fig. 1B).

## Extracellular polysaccharides are needed for biofilm formation

The complex regulatory networks described above converge on the operons needed for biofilm matrix production. The dominant exopolysaccharide required for biofilm formation is synthesized by the protein products of the 15 gene *epsA-O* operon (referred to as the *eps* operon). To date, only a subset of proteins encoded by the *eps* operon has been studied in any detail. EpsA and EpsB act as a tyrosine kinase modulator and tyrosine kinase, respectively, and both are required for biofilm formation (Gerwig *et al*., 2014). The target proteins of EpsB remain uncharacterized and additionally there are no obvious targets that can be elucidated from examination of global phosphoproteomic datasets (Levine *et al*., 2006; Macek *et al*., 2007; Elsholz *et al*., 2012). However, in combination with previous work showing that the tyrosine kinase modulator, TkmA and tyrosine kinase, PtkA affect biofilm formation (Kiley and Stanley-Wall, 2010), a role for tyrosine phosphorylation in modulating biofilm formation is fully supported. Given that EpsB and PtkA appear to have differing effects on complex colony architecture, it may be reasonable to hypothesize that each has distinct protein targets (Gerwig *et al*., 2014). It is likely that global phosphoproteomic analyses performed under biofilm formation conditions will be needed to identify the targets of these kinases that are involved in biofilm formation.

The bi-functional protein, EpsE, is the best characterized protein encoded by the *eps* operon (Blair *et al*., 2008; Guttenplan *et al*., 2010). EpsE can inhibit flagellar rotation by interacting with the flagellar rotor protein FliG (Blair *et al*., 2008; Guttenplan *et al*., 2010). EpsE is thought to function by directly interacting with a number of surface exposed residues on FliG (Blair *et al*., 2008). This prohibits the generation of torque, resulting in a lack of flagellar motility. Further to its role as a flagellar clutch, EpsE also acts as a glycosyltransferase enzyme to promote the production of the extracellular polysaccharide (Blair *et al*., 2008; Guttenplan *et al*., 2010). The two functions of EpsE are genetically separable as mutations in residues required for interaction with FliG do not perturb biofilm morphology, while mutation of the glycosyltransferase active site does not interfere with the ability of EpsE to abrogate motility (Guttenplan *et al*., 2010). The clutch activity of EpsE likely allows the cell to inhibit motility quickly and efficiently and synergizes with glycosyltransferase activity to promote biofilm formation (Blair *et al*., 2008; Guttenplan *et al*., 2010). Additionally, induction of *epsE* transcription to perturb flagellar rotation increases the DegU∼P level in the cell, leading to an increase in the transcription of *bslA* (Cairns *et al*., 2013) (Fig. 2). Consequently, EpsE provides a mechanism by which cells are able to inhibit flagellar motility and concurrently promote the synthesis of two distinct extracellular components needed for biofilm assembly.

## The composition of the biofilm polysaccharide

The chemical composition of the polysaccharide synthesized by the products of the *eps* operon is elusive and currently two contrasting monosaccharide analyses are available. When *B. subtilis* strain NCIB3610 is grown in a defined medium containing glutamic acid and glycerol, the monosaccharides present in the carbohydrate biomass are galactose, glucose and *N*-acetyl-galactose (GalNAc). The prevalence of each sugar was largely dependent on the integrity of the *eps* operon (Chai *et al*., 2012) and these findings are largely supported by unpublished data from the NSW laboratory. Consistent with these data, genes involved in galactose metabolism are important for biofilm formation (Chai *et al*., 2012). Contrastingly, analysis of the polysaccharide biomass generated by strain NCIB3610 grown in TY broth (LB medium supplemented with magnesium sulphate and manganese sulphate) revealed an *eps* operon dependent mannose-dominated profile of monosaccharides (Jones *et al*., 2014). Therefore the molecular nature of the polysaccharide produced by the components of the *eps* operon remains to be established and may depend on the substrates available.

In addition to the EPS produced using the products of the *eps* operon, strains of *B. subtilis* commonly used for the analysis of biofilm formation have the genetic capability to synthesize the extracellular polysaccharide levan. Levan is a homopolymer of fructose and production is dependent on the levansucrase encoded by *sacB* (Benigar *et al*., 2014). During growth in the presence of sucrose, levan can be incorporated into the matrix of the pellicle (Dogsa *et al*., 2013) and can partially compensate for the absence of the *eps* gene cluster. While levan is not essential for biofilm formation *in vitro*, on the basis that sucrose is produced by plants, the presence of levan in the matrix may be relevant for biofilm formation by *B. subtilis* in its natural environment in the rhizosphere (Dogsa *et al*., 2013). Therefore it is logical to deduce that the exopolysaccharides made by *B. subtilis* that contribute to the biofilm matrix are likely to vary with growth conditions.

## Extracellular proteins needed for biofilm formation

The main protein component of the biofilm matrix is TasA, which is encoded by the *tapA-sipW-tasA* operon (Branda *et al*., 2006). TasA was first defined as a spore-associated protein with antimicrobial activity (also named CotN) (Stover and Driks, 1999). However, subsequent analysis showed that deletion of *tasA* was associated with a lack of biofilm formation when rugose colony and pellicle morphology were assessed (Branda *et al*., 2006). The contribution of TasA to the matrix was found to be unique from that of the exopolysaccharide, as deletion of *tasA* produces a pellicle phenotype that is distinctive from that formed by an *eps* deletion strain (Branda *et al*., 2006). Moreover, co-culture of the two single (otherwise isogenic) deletion strains results in a wild-type biofilm phenotype (Branda *et al*., 2006), establishing that each product is a communal good that can be shared to benefit the entire population. TasA is localized to the biofilm matrix and its export from the cell is dependent on the SipW peptidase (Branda *et al*., 2006). Electron microscopy coupled with immunogold-labelling detection techniques showed that TasA forms fibres that extend from the cells. These findings, in combination with further experiments showing that TasA could polymerize *in vitro* and was able to bind to an antibody specific for intermediates of amyloid aggregates, led to the description of TasA as an amyloid-like protein (Romero *et al*., 2010). When TasA is purified from planktonic *B. subtilis* cells it is in an oligomeric state in solution. Fibre formation is stimulated by either a hydrophobic surface, such as electron microscopy grids (Romero *et al*., 2010), or an acidic solution (Chai *et al*., 2013). The secondary structure composition of TasA alters between its oligomeric and fibre states; in the oligomeric state the protein is α-helix rich, while during fibre formation a decrease in α-helices was observed along with a concurrent rise in β-sheet structure (Chai *et al*., 2013). This is a phenomenon that has been previously reported for several eukaryotic amyloid-like proteins [Alzheimer amyloid peptide a-β (Fraser *et al*., 1991), PI3 kinase (Zurdo *et al*., 2001) and a Syrian hamster prion protein (Sokolowski *et al*., 2003)].

The remaining protein encoded in the operon is TapA which forms a minor component of the TasA fibres and is required for their assembly (Romero *et al*., 2011; 2014[Bibr b68],[Bibr b69]). In the absence of *tapA*, not only are the wild-type TasA decorated fibres unable to form, but the level of TasA protein is also reduced (Romero *et al*., 2011). These data indicate that TapA is required for TasA stability and this may be due to the fibre form of TasA being more resistant to proteolytic cleavage than the unassembled oligomer. In agreement with these findings, phenotypically a *tapA* deletion strain is defective for pellicle formation (Romero *et al*., 2011). Interestingly, it has been identified that SipW, the peptidase required for secretion of both TapA and TasA to the matrix, is a bi-functional protein that has a second, and specific, role in the development of submerged surface-adhered biofilm communities formed by a laboratory isolate (Terra *et al*., 2012).

## The bacterial hydrophobin

An additional extracellular component needed for biofilm formation is the bacterial hydrophobin BslA (Kobayashi and Iwano, 2012; Hobley *et al*., 2013). BslA acts in a synergistic manner with both TasA and the EPS to allow biofilm assembly. This was concluded as deletion of *bslA* does not impact the synthesis of the high molecular weight polysaccharide or the generation of the TasA fibres synonymous with these matrix components, but does inhibit biofilm formation (Ostrowski *et al*., 2011). BslA is essential for both the observed complexity and the extreme hydrophobicity displayed by the mature biofilm (Kobayashi and Iwano, 2012; Hobley *et al*., 2013). While transcription of *bslA* is unimodal at the single cell level in the biofilm, BslA can be shared among non-producing members in a mixed strain biofilm (Hobley *et al*., 2013). BslA is a surface-active protein that forms a hydrophobic layer surrounding the colony biofilm and a ‘protein raft’ below the floating pellicle (Kobayashi and Iwano, 2012; Hobley *et al*., 2013). Consistent with these data, *in vitro* biophysical experiments demonstrated that BslA is capable of forming stable elastic films at hydrophilic to hydrophobic interfaces. Atomic level resolution of the BslA structure identified that BslA consists of two domains – an immunoglobulin-like domain and a unique highly hydrophobic ‘cap’ (Hobley *et al*., 2013). The hydrophobic cap is essential both for *in vivo* hydrophobicity of the biofilm and the stability of the *in vitro* elastic films formed by recombinant protein (Hobley *et al*., 2013). BslA has been termed a bacterial hydrophobin in homage to the fungal hydrophobins which form a hydrophobic protein coat on the surface of fungi (Elliot and Talbot, 2004), although, in actuality, similarities between BslA and the fungal hydrophobins are present at the physiochemical level and not the structural level. In short, BslA forms the third communal macromolecule made by *B. subtilis* during biofilm formation and further study is needed to elucidate the mechanisms underlying film formation and how BslA enables the assembly of the three dimensional biofilm.

## The function of the biofilm matrix

The extracellular matrix confers several properties that promote survival of *B. subtilis* in the biofilm. It allows the erection of aerial structures containing sporulating cells (Branda *et al*., 2001), confers extreme hydrophobicity (Epstein *et al*., 2011) (Fig. 1A), provides pressure to spread resident cells across a surface (Seminara *et al*., 2012) and is a source of mechanical stiffness (Asally *et al*., 2012; Wilking *et al*., 2013). The structure of the mature biofilm also serves a less obvious function and allows the formation of a network of liquid channels (Wilking *et al*., 2013) (Fig. 1B). Such a system facilitates distribution of nutrients to parts of the biofilm that would not be accessible using simple diffusion processes. The pressure within the channels is influenced by the rate of evaporation, driving liquid through the biofilm. The channels that form are interconnected and are maintained as the biofilm ages, indicating their physiological importance (Wilking *et al*., 2013). It is intriguing to speculate that the channels are lined by BslA to allow wicking of fluids into the deeper parts of the mature biofilm. Overall, the three dimensional architectural attributes that are imparted by the biofilm matrix are essential for the survival of its resident cells.

## Putting the wrinkles in the biofilm

Despite growing knowledge of the function of the biofilm matrix and its complex structure it is still not entirely clear how the wrinkles and ridges in the mature biofilm are generated. One possibility is that inherent elasticity of the extracellular matrix is sufficient (Trejo *et al*., 2013). This has been supported by biophysical experimentation in combination with theoretical analyses. Data suggested that mechanical buckling instability was responsible for shaping the biofilm; i.e. when the cells in the biofilm push against a surface wrinkles are induced. The matrix is essential for this to occur as it confers elastic properties to the biofilm (Trejo *et al*., 2013). Additionally, it has been highlighted that localized cell death is involved in wrinkle formation (Asally *et al*., 2012). Indeed, cell death at the base of the biofilm was linked with buckling in the vertical plane and thus wrinkles. The mechanical strength of the matrix was needed for this to occur as deletion of genes associated with matrix production, including *epsH* and *tasA*, resulted in a more homogenous pattern of cell death that altered biofilm architecture to the extent that wrinkles were not formed. Interestingly, it was shown that wrinkles were formed in patterns that mirrored cell death zones when cell density was artificially increased in localized areas to increase cell death. This could indicate that wrinkling might allow the dissipation of mechanical forces that occur due to high cell density and subsequent cell death (Asally *et al*., 2012). It is highly likely that mechanical forces, potentiated by as yet undefined specific interactions between the extracellular molecules of the matrix, and localized cell death combine to allow the beautiful and intricate wrinkles to evolve over time.

## Disassembly of the biofilm

The final stage of the biofilm cycle is that of disassembly. There are at least two mechanisms by which this could occur. First, transcription of the operons required for biofilm matrix production could be silenced and second the macromolecules in the biofilm matrix could be disrupted or degraded. We will first examine the potential for gene silencing. As discussed above matrix gene expression is bimodal in the population and is subject to hysteresis that locks cells into a state where the biofilm matrix is synthesized (Vlamakis *et al*., 2013). It has however been shown that some cells switch off matrix production and can return to a motile cell state (Vlamakis *et al*., 2008; Norman *et al*., 2013). At the level of gene expression this would involve reversing the SlrR-high state back to a SlrR-low state. In line with this, it has been noted that SlrR levels decline as the biofilm matures, a phenotype that was tied to instability of the protein (Chai *et al*., 2010a). The instability of SlrR was attributed to two underlying mechanisms: (i) cleavage by the ClpCP protease and (ii) autocleavage (Chai *et al*., 2010a). SlrR was identified as having a conserved motif usually found in LexA-type repressors, which undergo autocleavage upon the sensing of a cellular signal (Little, 1984). Indeed, site-directed mutation of amino acids in this motif resulted in increased stability of SlrR. However, while SlrR carries a LexA-type motif, it does not have the catalytic domain that would be essential for proteolytic activity (Newman and Lewis, 2013). Therefore, an alternative model has been presented where it is proposed that, due to the presence of two helical hooks, SlrR is able to aggregate which would result in its proteolytic cleavage by ClpCP (Newman and Lewis, 2013). The destruction of SlrR would allow SinR to engage with the matrix promoter regions thereby shutting down biosynthesis of TasA and the exopolysaccharide.

The second method of biofilm dispersal would involve degradation or disruption of the macromolecules in the extracellular environment. Consistent with this mechanism of dispersal, production of extracellular proteases has been correlated with late stages of biofilm formation (Marlow *et al*., 2014a). While it is important to keep in mind that a functional role for proteases in breaking down the protein components of the biofilm matrix has not yet been established, such activity is supported by the knowledge that the protease nattokinase of *B. subtilis* Natto has amyloid-degrading capabilities (Hsu *et al*., 2009). Therefore, it can be postulated that such an enzyme could have a role in disassembly of the TasA amyloid-like fibres in the matrix. Two further mechanisms of disassembly at the level of macromolecule hindrance have been proposed. The first was based on self-production of d-amino acids by late-stage biofilms (Kolodkin-Gal *et al*., 2010). d-amino acids were hypothesized to trigger disassembly by incorporating into the peptidoglycan cell wall and blocking TapA embedding into the wall, resulting in the release of the TasA fibres from the cell (Kolodkin-Gal *et al*., 2010; Romero *et al*., 2011). However, a recent study disputes this mechanism as it was elucidated that addition of d-amino acids resulted in misincorporation of d-amino acids into proteins, which reduced cellular growth (Leiman *et al*., 2013). Incorporation of d-amino acids into proteins can be prevented in the presence of a functional d-aminoacyl-tRNA deacylase that removes d-amino acids from mischarged tRNAs (Soutourina *et al*., 2004). The *B. subtilis* strain used in the initial analysis contained a mutation in the d-aminoacyl-tRNA deacylase gene (namely *dtd*) that prevented expression of this enzyme (Leiman *et al*., 2013). When the mutation was repaired the d-amino acid inhibition of biofilm formation was not observed (Leiman *et al*., 2013). The second proposed mechanism of biofilm disassembly was self-production of the polyamine norspermidine (Kolodkin-Gal *et al*., 2012). It was postulated that within the extracellular environment norspermidine interacts with the exopolysaccharide component of the biofilm matrix, collapsing it and releasing the cells (Kolodkin-Gal *et al*., 2012). However, further studies have shown that *B. subtilis* completely lacks the biosynthetic pathway required for norspermidine synthesis and, consistent with this, detection of norspermidine within biofilm samples was not possible despite utilization of two separate detection methods (Hobley *et al*., 2014). Furthermore, analysis indicated that heterologous addition of low concentrations of norspermidine can replace the role of the related and natively produced polyamine, spermidine, during biofilm formation (Burrell *et al*., 2010; Hobley *et al*., 2014). It is therefore safe to say that the issue of biofilm disassembly by *B. subtilis* remains a much-debated topic within the field. Further investigation will be required to determine whether the reduction in biofilm biomass observed in late-stage biofilms is the result of an organized disassembly process or simply the result of the onset of sporulation by the majority of the population after exhaustion of the nutrient supply.

## Looking forward

The environmental signals and regulatory pathways that control entry into biofilm formation have been well studied and it is known that they largely converge to control the production of the biofilm matrix components (Vlamakis *et al*., 2013). Regulation of transcription is critical to biofilm formation as it allows deployment of the matrix molecules at the correct time, and in the correct place. However, what is less understood is how the macromolecules interact in the extracellular environment to provide structure and rigidity, and moreover how they interact with surfaces in the host environment. It is likely that our understanding of this area of biofilm biology will require interdisciplinary collaborations as the techniques needed to illuminate this black box of biology will draw on carbohydrate chemistry, surface chemistry, and biophysics in combination with molecular biology. However enhanced knowledge in this arena is likely to be profitable as understanding the molecular nature of the biofilm matrix interactions will be a prelude to promoting or disrupting biofilm formation within healthcare, agricultural and industrial settings.
